# Perception of effort and the allocation of physical resources: A generalization to upper-limb motor tasks

**DOI:** 10.3389/fpsyg.2022.974172

**Published:** 2023-01-24

**Authors:** Marie Payen de la Garanderie, Aymeric Courtay, Camille Féral-Basin, Pierre Rainville, Jérémie Gaveau, Benjamin Pageaux

**Affiliations:** ^1^École de Kinésiologie et des Sciences de l’Activité Physique (EKSAP), Faculté de Médecine, Université de Montréal, Montreal, QC, Canada; ^2^Centre de Recherche de l’Institut Universitaire de Gériatrie de Montréal (CRIUGM), Montreal, QC, Canada; ^3^INSERM UMR1093-CAPS, Université Bourgogne Franche-Comté, UFR des Sciences du Sport, Dijon, France; ^4^Département de Stomatologie, Faculté de Médecine Dentaire, Université de Montréal, Montreal, QC, Canada; ^5^Espace d’Etude du Mouvement—Etienne Jules MAREY, Université Bourgogne Franche-Comte, UFR des Sciences du Sport, Dijon, France; ^6^Centre Interdisciplinaire de Recherche sur le Cerveau et l’Apprentissage (CIRCA), Montreal, QC, Canada

**Keywords:** perceived exertion, upper-limb task, CR100 scale, motor control, psychophysiology, box and block test, pointing tasks

## Abstract

**Purpose:**

The perception of effort (PE) is widely used to prescribe and monitor exercise during locomotor and resistance tasks. The present study examines the validity of PE to prescribe and monitor exercise during upper-limb motor tasks under various loads and speed requirements.

**Methods:**

Forty participants volunteered in two experiments. In experiment 1, we used four PE intensities to prescribe exercise on a modified version of the box and block test (BBT) and a pointing task. We investigated the possibility of monitoring the exercise intensity by tracking changes in PE rating in response to three different tempos or additional weights. Experiment 2 replicated the possibility of prescribing the exercise with the PE intensity during the BBT and explored the impact of additional weights on performance and PE during the standardized version of the BBT. Muscle activation, heart rate, and respiratory frequencies were recorded.

**Results:**

In experiment 1, increasing the PE intensity to prescribe exercise induced an increased performance between each intensity. Increasing task difficulty with faster movement tempo and adding weight on the forearm increased the rating of PE. Experiment 2 replicated the possibility to use PE intensity for exercise prescription during the BBT. When completing the BBT with an additional weight on the forearm, participants maintained performance at the cost of a higher PE. In both experiments, changes in PE were associated with changes in muscle activation.

**Conclusion:**

Our results suggest that PE is a valid tool to prescribe and monitor exercise during upper-limb motor tasks.

## 1. Introduction

The perception of effort, also known as perceived exertion or sense of effort (Marcora, 2010; Pageaux, 2016), can be described as “*the particular feeling of that energy being exerted*,” and “*is accompanied by a sensation of strain and labor, a feeling that intensifies the harder a person tries*” (Preston and Wegner, 2009). Effort is experienced during physical (e.g., running to catch the bus) or cognitive tasks (e.g., completing Sudoku) and in the context of self-restraint behavior (e.g., smoking cessation; Preston and Wegner, 2009). It is thought to influence how we move, i.e., how the nervous system selects a given movement among a myriad of possibilities (Izawa et al., 2008; Gaveau et al., 2021). Due to its omnipresence in our daily life, the interest in understanding the perception of effort is growing among researchers. This perception is linked to the task intensity and the amount of resources invested (Inzlicht et al., 2018); strongly influences the self-regulation of human behavior (Marcora, 2015; Inzlicht et al., 2018); is one of the main features of fatigue in various contexts (Enoka and Stuart, 1992; Pageaux and Lepers, 2016); and is exacerbated in various pathologies such as chronic fatigue syndrome (Cook et al., 2007; Barhorst et al., 2020), stroke (Kuppuswamy et al., 2015), chronic kidney disease (Macdonald et al., 2012), or cancer (Fernandez et al., 2020). Perception of effort is a fundamental experience that directly influences our everyday decisions to engage or disengage in various actions, by monitoring the cognitive and motor resources necessary to perform any task (Preston and Wegner, 2009; Pageaux, 2016). The perception of the amount of effort invested in a task is also closely linked to the regulation of motor performance (Pageaux, 2014, 2016; Marcora, 2019). According to the motivation intensity theory (Brehm and Self, 1989; Richter et al., 2016), one maintains performance by increasing effort when task difficulty increases and one lets performance decrease when no longer able or willing to invest additional effort.

Perception of effort is widely investigated during global locomotor tasks, such as walking or cycling, in both healthy and symptomatic populations (Horstman et al., 1979; Au et al., 2017; Zinoubi et al., 2018; Décombe et al., 2020; Flairty and Scheadler, 2020) to prescribe and monitor exercise (Impellizzeri et al., 2004; Azevedo et al., 2016; Eston and Parfitt, 2018). Perception of effort is also investigated during isolated motor tasks involving the upper or lower limb, in strength training programs (Miller et al., 2009; Zourdos et al., 2016), in studies aiming at better understanding the regulation of endurance performance (Maikala and Bhambhani, 2006; Pageaux et al., 2013) or the mechanisms associated with the development of muscle fatigue during repetitive tasks (de Morree et al., 2012; Otto et al., 2019; Yang et al., 2019; Jacquet et al., 2021). To the best of our knowledge, most of the studies investigating the perception of effort are performed during locomotor exercises or isolated exercises performed with the lower limbs (de Morree et al., 2014; Meir et al., 2015; Luu et al., 2016; Faelli et al., 2019). Although the perception of effort is of interest to understand how the nervous system controls our everyday movements, motor control studies mostly indirectly investigated it by measuring the force output, the decision made by the participants or motor strategies (Izawa and Shadmehr, 2008; Shadmehr et al., 2016; Cos, 2017; Morel et al., 2017; Gaveau et al., 2021; Wang et al., 2021). While these methods present several advantages in the context of decision-making tasks, not considering the rating of perception of effort as a dependent variable limits the exploration of the subjective experience of the participant during task completion (Pageaux, 2016; Wang et al., 2021). As the perception of effort has been recently proposed to finely regulate motor control (Cos, 2017) and, thus, to affect decision-making and performance in a task involving movement regulation (Shadmehr et al., 2016; Wang et al., 2021), there is an urgent need for studies exploring the perception of effort during upper limb tasks. Such studies could provide opportunities to better understand the interaction between the perception of effort and motor control.

In this context, the present study aimed to validate the use of the perception of effort to prescribe and monitor exercise in healthy young adults performing upper limb motor tasks. To do so, two experiments manipulated the physical demand to alter the task difficulty. In the first experiment, by using a modified version of the classical box and block test (Mathiowetz et al., 1985) and a pointing task, we tested the possibility (i) to prescribe exercise at different intensities with the perception of effort and (ii) to monitor changes in perception of effort when task difficulty was altered with manipulation of the physical demand. As effort and its perception vary in relation to performance (Brehm and Self, 1989; Richter et al., 2016), we monitored the perception of effort while controlling for performance. We hypothesized that (i) it is possible to prescribe different exercise intensities with the perception of effort, as attested by an increased task performance when the prescribed intensity of perceived effort increases and (ii) increasing task difficulty, with faster tempos or additional weights, will be reflected in higher perceptions of effort. In the second experiment, by using the classical box and block test with its validated instructions, we tested the effect of increasing physical demand on subsequent performance and rating of perception of effort. We hypothesized that performance could be maintained at the cost of a higher resource mobilization as reflected by the increases in the perception of effort.

## 2. Materials and methods

### 2.1. Participants

Twenty participants volunteered to participate in experiment 1 and twenty participants volunteered to participate in experiment 2. The description of the participants is available in Table 1. None of the participants reported any pain-related, neurological, psychological disorders, or somatic illnesses. Written informed consent was obtained from each participant. Experiment 1 took place at the Centre de recherche de l’Institut universitaire de gériatrie de Montréal. Experiment 2 took place at the Espace d’Etude du Mouvement—Etienne Jules MAREY de l’Université de Bourgogne. We performed two experiments with different participants to challenge the replication of our results. All participants gave written informed consent, and procedures were approved by the local ethics committee (CER VN 18-19-35). As caffeine and sleep deprivation are known to alter the perception of effort (Temesi et al., 2013; de Morree et al., 2014), participants in both experiments were asked to refrain from ingesting caffeine at least 3 h before their visits and to get at least 7 h of sleep the night before.

**TABLE 1:** Description of participants.

	Experiment 1		Experiment 2	
	**Women (*n* = 18)**	**Men (*n* = 2)**	**Women (*n* = 7)**	**Men (*n* = 13)**
Age (yrs)	24 ± 2	24 ± 2	26 ± 2	25 ± 2
Weight (kg)	62 ± 11	72 ± 14	59 ± 7	76 ± 10
Height (cm)	164 ± 10	187 ± 5	163 ± 6	178 ± 5.4
Physical activity (/30)	19.06 ± 5.4	23 ± 0	21.5 ± 6.3	23.6 ± 3.5
Right-handed	17	2	7	11
Left-handed	1	–	–	2

Yrs, years; kg, kilogram; cm, centimeter. The physical activity score was measured with the Dijon physical activity questionnaire (Robert et al., 2004). Data are presented as mean ± SD.

### 2.2. Upper limb motor tasks

In this study, the upper limb motor tasks were the Box and Block Test (BBT) and a Pointing Task (PT). A full description of these tests is available below. We chose these two tests for their relevance in the context of clinical settings as well as research.

#### 2.2.1. Box and block test

**FIGURE 1:**
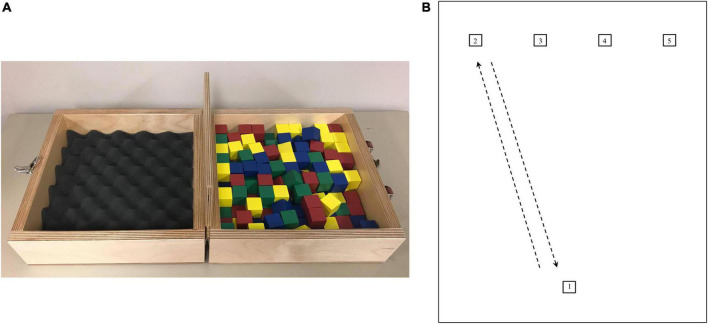
**(A)** Illustration of the box and block test (Mathiowetz et al., 1985) used in experiments 1 and 2. Briefly, participants had to grasp one block at a time with the dominant hand, transport the block over the partition, and release it into the opposite compartment. **(B)** Illustration of the pointing task used in experiment 1. Starting from target 1, participants had to go back and forth between each target. Right-handed participants started by reaching target 2 for their first-round trip, while left-handed participants started by reaching target 5 for their first-round trip. Measures are being taken from the center of all squares (1 × 1 cm). The distance between each upper square is 5.1 cm. The distance between targets 1–2 and 1–5 is 22.3 cm, respectively. The distance between targets 1–3 and 1–4 is 21 cm, respectively.

The BBT (Mathiowetz et al., 1985), illustrated in Figure 1A, is used to assess manual dexterity, defined as “the ability to make coordinated hand and finger movements to grasp and manipulate objects” (Makofske, 2011). This test has been validated in several populations such as older adults (Desrosiers et al., 1994), fibromyalgia patients (Canny et al., 2009), and stroke rehabilitation (Lin et al., 2010). The test consists of a wooden box (53.7 cm × 25.4 cm × 8.5 cm) separated into two containers of 25.4 cm each. It includes 150 wooden cubes (2.5 cm). Participants have to grasp one block at a time with the dominant hand, transport the block over the partition, and release it into the opposite compartment. Standardized instructions require participants to move as many blocks as possible in 60 s, and performance is monitored as the number of blocks moved. In experiment 1, we used a 30-s modified version of the BBT where participants had to move the blocks at a prescribed effort intensity or by following a pre-determined tempo signaled by an auditory cue to control for the number of blocks moved (performance). In experiment 2, we used the standardized instructions in the absence and presence of additional weight on the dominant forearm. In both experiments, the compartment containing the block was placed in front of the participants’ dominant hand. Errors were visually counted by an experimenter when the fingertips did not go beyond the partition, and the associated block was not counted in the final score. Participants were informed that blocks will not be counted in the final score when the fingertips do not go beyond the partition.

#### 2.2.2. Pointing task

Pointing tasks (PT) are widely used in research to study motor control (e.g., Domkin et al., 2002; Missenard et al., 2009). A PT (illustrated in Figure 1B) was performed in experiment 1. Participants had to go back and forth between targets (squares of 1 cm^2^) as quickly as possible in a given time. Participants started from target 1 (reference target) and had to follow a pre-determined order, depending on their dominant hand. Right-handed participants had to reach target 2 and come back to target 1, then reach target 3 and come back to target 1, then reach target 4 and come back to target 1, and then reach target 5 and come back to target 1. This sequence was repeated for 30 s, either with the instructions of reaching the targets at a prescribed effort intensity or by following a pre-determined tempo to control for the number of targets reached (i.e., performance). For left-handed participants, the order of the sequence was reversed. They had to first reach target 5. Participants performed the test with a pencil in their hand and had to point where they reached, thus allowing an experimenter to visually control for the exact number of targets correctly reached. Participants were informed that a target will be counted in the final score only when the mark is placed inside a target.

### 2.3. Overview of the two experiments

#### 2.3.1. Experiment 1

**FIGURE 2:**
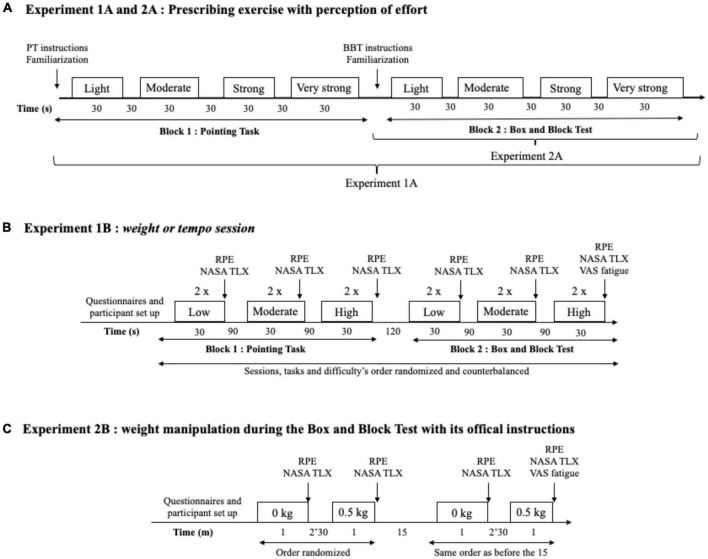
**(A)** Experiments 1A and 2A overview: The procedures used to test the possibility to prescribe exercise using the perception of effort. The exercise was prescribed at four intensities of perceived effort *via* the CR100 scale: light (13/100), moderate (23/100), strong (50/100), and very strong (70/100). While both the pointing task (PT) and the box and block test (BBT) were performed in experiment 1A, only the BBT was performed in experiment 2A. **(B)** Experiment 1B overview. Set up consisted of the placement of the respiratory frequency belt, heart rate monitor, and EMG surface electrodes. Then, participants completed the indicated questionnaire or visual analog scale (VAS). Participants performed two repetitions per level of difficulty with 30 s of recovery in between. Rating of perceived effort (RPE) and subjective workload using NASA TLX scale were assessed in-between each level of difficulty. **(C)** Experiment 2B overview. Participants performed the box and block test for 60 with the absence (0 kg) or presence (0.5 kg) of additional weights. Set up consisted of the placement of the heart rate monitor and the EMG surface electrodes. Then, participants completed the indicated questionnaire or scale.

This experiment aimed to test, with a modified version of the BBT and a PT, the possibility (i) to use the perception of effort to prescribe exercise (Exp. 1A), and (ii) to monitor changes in the rating of perception of effort when performance is controlled, and task difficulty manipulated (Exp. 1B). (i) To test the possibility of prescribing exercise with a target level of perceived effort, we monitored performance associated with four intensities of perception of effort (presented in Figure 2A). (ii) To test the possibility of monitoring changes in the perception of effort, we manipulated task difficulty by increasing physical demand. Task difficulty was increased by increasing the speed of movement (*tempo session*) or by adding a weight on the forearm (*weight session*). The weight session was performed at a controlled pace such that the effect of task demand on perception of effort was assessed at a controlled performance level (i.e., constant speed). The *tempo session* and *weight session* were performed in two different laboratory visits, in a randomized order. An overview of the sessions is presented in Figure 2B. All tests were performed in a seated position. At the onset of the first laboratory visit, participants completed several questionnaires allowing the characterization of the population studied (anthropometry, physical activity score; Robert et al., 2004), Edinburgh Handedness Inventory (Oldfield, 1971). Then, each session was performed as described below, with all BBT trials performed in one block and all PT trials related performed in another block. The order of each block (BBT performed first vs. PT performed first) was randomized between participants and kept constant for each participant between the two laboratory visits (*tempo session* vs. *weight session*).

##### 2.3.1.1. Tempo session

Participants were equipped with the apparatus allowing measurement of EMG, heart rate, and/or respiratory frequency. We subsequently provided standardized instructions on how to use the psychophysical rating scale to monitor the perception of effort and how to perform the BBT and the PT. Participants had 1 min to familiarize themselves with each test and could ask any questions. Following this familiarization, participants were asked to perform a block of trials for the BBT or PT. The first block consisted of trials using a target level of perceived effort intensity to prescribe the exercise, and the second block consisted of trials where performance was controlled by different tempos and where the perception of effort was reported by the participant. Trials related to the use of perception of effort to prescribe the exercise intensity consisted of performing one test of 30 s per target perceived effort intensity level (light effort, moderate effort, strong effort, and very strong effort), with each test interspaced by 30 s of recovery. The experimenter recorded performance for each prescribed intensity. Then, participants performed two tests of 30 s per difficulty level (low, moderate, and high), with each test interspaced by 90 s of recovery. Once a block (BBT vs. PT trials) was completed, a 120 s rest was given, and participants completed the other block following the same structure. Following pilot experiments, three tempos specific to each task were chosen to produce three levels of difficulty. For the PT, the following tempos were used: 1 Hz (slow tempo), 1.5 Hz (moderate tempo), and 2 Hz (fast tempo). For the BBT, the following tempos were used: 0.5 Hz (slow tempo), 0.75 Hz (moderate tempo), and 1 Hz (fast tempo). The order of the level of difficulties was randomized. The rating of perceived effort was measured immediately at the end of each repetition. Following the two repetitions of each of the difficulty level, participants reported their perceived workload using the NASA TLX scale as described below.

##### 2.3.1.2. Weight session

The procedures in the *weight sessions* are identical to the procedures in the *tempo session*, except that task difficulty was manipulated by adding weights (4-lb pair, Enhance Fitness) on the dominant forearm of the participant while performing the BBT and PT at a fixed tempo (BBT: 0.75 Hz; PT: 1.5 Hz). The low difficulty level was performed with no additional weight (0 kg, light weight) on the forearm. The moderate and high difficulty levels were performed with additional weights, 0.5 kg (moderate weight) and 1 kg (heavy weight), respectively, on the forearm.

#### 2.3.2. Experiment 2

The second experiment aimed (Exp. 2A) to replicate the results of the perception of effort prescription condition of experiment 1A and to test the effect of increasing physical demand to manipulate BBT difficulty on subsequent performance and ratings of perception of effort (Exp. 2B). Participants visited the laboratory one time. At their arrival, participants were equipped with the apparatus allowing measurement of EMG and heart rate. We subsequently provided standardized instructions on how to use the psychophysical scale to monitor the perception of effort and how to perform the BBT. Participants had 1 min to familiarize themselves with each test and could ask any questions. Following this familiarization, participants were asked to perform two blocks of trials. The first block consisted of trials related to using the perception of effort intensity to prescribe the exercise, as performed in experiment 1. In the second block of trials, participants completed the BBT according to the standardized duration of 60 s, in the absence (0 kg, low difficulty level) and the presence (0.5 kg, high difficulty level) of additional weight on the dominant forearm, interspaced by a 2.5 min recovery between difficulties. The order of difficulty levels (0 kg, low difficulty level vs. 0.5 kg, high difficulty level) was randomized between participants and repeated after a 15 min break. In total, each participant repeated each level of difficulty twice.

Pilot experiments revealed that the duration of 60 s with an additional weight of 1 kg induced an important level of fatigue in the participants. Consequently, to limit the induction of fatigue, the high level of difficulty was performed with a weight of 0.5 kg and a between level of difficulty recovery period of 2.5 min. The rating of perceived effort and performance (i.e., number of blocks moved) was monitored immediately at the end of each repetition (three repetitions per level of difficulty, with the order of difficulty randomized). Following each level of difficulty, participants reported their perceived workload using the NASA TLX scale as described below. An overview of the session is presented in Figure 2C.

### 2.4. Psychological measurements

#### 2.4.1. Perception of effort

Perception of effort, defined as the conscious sensation of “how hard, heavy and strenuous a physical task is” (Marcora, 2010; Pageaux, 2016), was measured and used to prescribe the exercise with the CR100 scale (Borg and Kaijser, 2006). This scale ranges from 0 (“nothing at all”) to 100 (“maximal”) and includes verbal anchors, such as light (weak), moderate, and strong (heavy) for intermediate values (Borg and Kaijser, 2006). Standardized instructions on how to use the CR100 scale were provided. Then, participants received standardized instructions on how to evaluate the perception of effort and exclude the perception of pain from their rating (Pageaux, 2016; Pageaux et al., 2020). Participants had the opportunity to ask questions on the scale and effort rating instructions before starting the experiments. To prescribe exercise, participants were asked to perform the tasks at four different effort intensities associated with the following verbal anchors and numbers on the CR100 scale: light (13), moderate (23), strong (50), and very strong (70). To report their perception of effort, participants were asked to first refer to the verbal anchors and then to report a number that best represents the intensity of their perception. The CR100 scale was printed in a legal format (8.5 × 14 in) and fixed on a wall ∼1 m in front of the participants.

#### 2.4.2. Perceived workload

Perceived workload was measured with the Nasa Task Load Index (NASA TLX; Hart and Staveland, 1988). In line with the aims of our study, only the four following subscales were considered: Physical Demand, Mental Demand, Temporal Demand, and Effort. Participants had to score each of the items on a scale divided into 20 equal intervals anchored by a bipolar descriptor (e.g., High/Low). This score was multiplied by 5, resulting in a final score between 0 and 100 for each of the six subscales.

#### 2.4.3. Fatigue

The presence of fatigue is known to increase the perception of effort (Enoka and Stuart, 1992; Pageaux and Lepers, 2016). We consequently monitored feelings of fatigue at the beginning and the end of each visit with a visual analog scale (Le Mansec et al., 2017). Participants had to place a mark on a 100 mm line with bipolar end anchors (0 = not fatigued at all; 100 = extremely fatigued). The fatigue score was determined by measuring the distance (in mm) from the left-hand end of the line to the mark made by the participant.

### 2.5. Physiological measurements

***Electromyography*** (EMG) of the biceps brachii and triceps lateral head was measured in both experiments with adhesive, pre-gelled surface electrodes (Covidien, CA). The decision to measure muscle activation of these two muscles was taken following a preliminary experiment where participants (*N* = 20) performed the block and block tests with and without the addition of a 0.5 kg weight on the forearm. During task completion, measurements of the EMG signal of eight muscles were performed. The results are available in Supplementary material and revealed that the biceps brachial was the muscle presenting the greater increase in root mean square EMG in the presence of the additional weight over the forearm. Consequently, we decided to measure as a second muscle an antagonist, the triceps lateral head. Before placing the electrodes, the skin was shaved, cleaned with alcohol, and dried. Electrodes were placed using SENIAM recommendations (Hermens et al., 2000). The electrode reference was attached to the extremity of the elbow of the dominant arm. In experiment 1, EMG was recorded using a PowerLab system (26T, ADInstruments) with an acquisition rate of 1 KHz and filtered with bandpass ranging from 20 to 400 Hz (auto adjust) and a notch filter with a center frequency of 60 Hz (auto adjust). Data were analyzed using the LabChart software (AD Instruments). In experiment 2, EMG was recorded using a Biopac system (MP150, Biopac Systems, Inc.) with an acquisition rate of 1 KHz and filtered with bandpass ranging from 20 to 400 Hz (auto adjust) and a notch filter with a center frequency of 60 Hz (auto adjust). Data were analyzed using Acknowledge software (Biopac Systems, Inc.). The root mean square (RMS) was automatically calculated with each software. Data were averaged for the last 5 s of each 30 s (experiment 1) or 60 s (experiment 2) trials.

***Heart rate frequency*** was measured in both experiments. In experiment 1, we used a finger pulse transducer (TN1012/ST, AD Instruments) placed on the non-dominant index finger. To limit movement artifacts, the non-dominant hand was placed on a homemade support to rest on the table and stay as steady as possible. The signal was recorded with an acquisition rate of 1 KHz and filtered with a digital filter of 7 Hz (low pass). Data analysis was automatically performed by the LabChart software. Heart rate frequency was averaged for the last 5 s of each 30 s trials. Due to numerous movement artifacts in experiment 1, monitoring heart rate was measured using a chest strap *via* the paired Polar watch (Polar RS400; Polar Electro Oy, Kempele, Finland) and measured as the average of the 60 s trial. The experimenter pressed the start/stop button of the watch at the beginning and end of each trial and then recorded the average heart rate frequency calculated by the watch.

***Respiratory frequency*** was measured in experiment 1 only *via* a respiratory belt transducer (TN11132/ST, AD Instruments). The respiratory belt was fixed on the participant’s chest, the signal was recorded with an acquisition rate of 1 KHz and filtered with a digital filter of 7 Hz (low pass). Data analysis was automatically performed by the LabChart software. Respiratory frequency was averaged from the last 5 s of each 30 s trials.

### 2.6. Statistical analysis

All data are presented as mean ± standard deviation in the text. Assumptions of statistical tests such as normal distribution and sphericity of data were checked as appropriate. Greenhouse-Geisser correction to the degrees of freedom was applied when violation to sphericity was present.

#### 2.6.1. Experiment 1A

All analyses subsequently described were performed for the modified BBT and PT. A 2 × 4 repeated-measures ANOVA was used to assess the effects of visits (1 and 2) and effort intensity (light, moderate, strong, and very strong) on performance, heart rate frequency, and respiratory frequency. A 2 × 4 × 2 repeated-measures ANOVA was used to assess the effects of visit (1 and 2), effort intensity (light, moderate, strong, and very strong), and muscle (biceps brachial and triceps brachial) on RMS EMG. As these analyses were performed to test the possibility to use the perception of effort to prescribe the exercise, a significant main effect of effort intensity only was followed with the following pairwise comparisons adjusted with the Bonferroni correction: light effort vs. moderate effort, moderate effort vs. strong effort, and strong effort vs. very strong effort.

#### 2.6.2. Experiment 1B

To test the possibility to monitor changes in perception of effort when task difficulty is altered with manipulation of the physical demand in both *tempo* and *weight sessions*, a repeated-measures ANOVA was used to assess the effects of difficulty (easy, medium, and hard) on heart rate and respiratory frequencies. A 3 × 2 repeated-measures ANOVA was used to assess the effects of difficulty (easy, medium, and hard) and muscle (biceps brachial and triceps brachial) on RMS EMG. The significant effect of difficulty was followed-up with pairwise comparisons adjusted with the Bonferroni correction. A Friedman ANOVA was used to assess the effects of difficulty on performance, rating of perceived effort, as well as the physical demand, mental demand, temporal demand, and effort subscales of the NASA TLX scale. The significant effect of difficulty was followed up with the Wilcoxon signed-ranked tests adjusted with the Bonferroni correction.

#### 2.6.3. Experiment 2A

A repeated-measures ANOVA was used to assess the effects of effort intensity (light, moderate, strong, and very strong) on performance, heart rate frequency, and RMS EMG. A 4 × 2 repeated-measures ANOVA was used to assess the effects of effort intensity (light, moderate, strong, and very strong) and muscle (biceps brachial and triceps brachial) on RMS EMG. As these analyses were performed to test the possibility to use the perception of effort to prescribe the exercise, a significant effect of effort intensity only was followed with the following pairwise comparisons adjusted with the Bonferroni correction: light effort vs. moderate effort, moderate effort vs. strong effort, and strong effort vs. very strong effort.

#### 2.6.4. Experiment 2B

A 2 × 2 repeated-measures ANOVA was used to assess the effects of repetition (1 and 2) and difficulty (easy and hard) on performance, rating of perceived effort, heart rate frequency, as well as the physical demand, mental demand, and effort subscales of the NASA TLX scale. A 2 × 2 × 2 repeated-measures ANOVA was used to assess the effects of repetition (1 and 2), difficulty (easy and hard), and muscle (biceps brachial and triceps brachial) on RMS EMG. As experiment 2B did not constrain the temporal demand of the task by imposing a tempo, we did not analyze the temporal demand subscale of the NASA TLX scale. If a repetition × difficulty interaction reached significance, the following follow-up tests were performed and adjusted with the Bonferroni correction: repetition 1/0 kg vs. repetition 2/0 kg, repetition 1/0.5 kg vs. repetition 2/0.5 kg, repetition 1/0 kg vs. repetition 1/0.5 kg, and repetition 2/0 kg vs. repetition 2/0.5 kg.

**For both experiments**, all statistical analyses were performed using the Statistical Package for the Social Sciences software, version 27 for Mac OS X (SPSS, Chicago, IL) and jamovi software, version 2.0.0.0. Effect sizes for the repeated measures ANOVA are reported as the partial eta squared (η_*p*_^2^) provided by SPSS. Effects sizes for the pairwise comparisons are reported with *r* and calculated with Microsoft Excel according to the equations described below for parametric (i) and non-parametric and (ii) tests (Field, 2005). Parameters *t*, *df*, and *Z* were provided by SPSS, and *N* corresponds to the total number of observations (Field, 2005).


$$\left(i\right)\,r=\sqrt{\frac{t^{2}}{t^{2}+d\,f}}\;\left(\mathrm{ii}\right)\,r=\frac{Z}{\sqrt{N}}$$

Significance was set at 0.05 (2-tailed). Thresholds for small, moderate, and large effects were set at 0.1, 0.3, and 0.5 for *r* (Cohen, 1988).

## 3. Results

### 3.1. Experiment 1

In this experiment, we used a modified version of the BBT and PT. We prescribed 30 s of exercise performed at four intensities of effort (light, moderate, strong, and very strong) in two different visits. Performance, RMS EMG, heart rate, and respiratory frequencies were monitored for each prescribed effort intensity. We also manipulated task difficulty levels (low, moderate, and high) by manipulating physical demand and imposing three tempos or adding three different weights on the participant’s dominant forearm while performing the task at a fixed tempo. Performance, heart rate frequency, respiratory frequency, RMS EMG, and the subjective workload were measured for each difficulty.

#### 3.1.1. Experiment 1A: Using the perception of effort to prescribe the exercise

**FIGURE 3:**
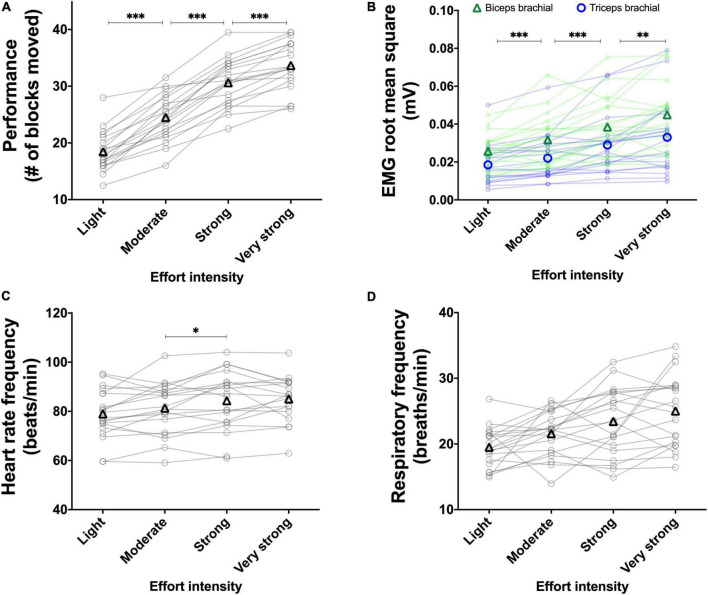
Experiment 1A: Using the perception of effort to prescribe the exercise during the box and block test. Effect of increasing the prescribed effort intensity on performance (**A**, *n* = 20), EMG root mean square of the biceps (green line) and triceps (blue line) brachial muscles (**B**, *n* = 19), heart rate frequency (**C**, *n* = 18), and respiratory frequency (**D**, *n* = 20) during the box and block test. The exercise was prescribed at four intensities of perceived effort via the CR100 scale: light (13/100), moderate (23/100), strong (50/100), and very strong (70/100). Data are presented as the main effect of effort intensity **(A, C, D)** and effort intensity × muscle interaction **(B)**. The n indicates the number of participants with all the data in each four effort intensities. Changes in the n reflect data loss due to the issue with equipment or movement artifact. Individual data are presented in light markers and means in dark markers. *Main effect of intensity, the difference between two effort intensities. *p < 0.05, **p < 0.01, and ***p < 0.001.

**FIGURE 4:**
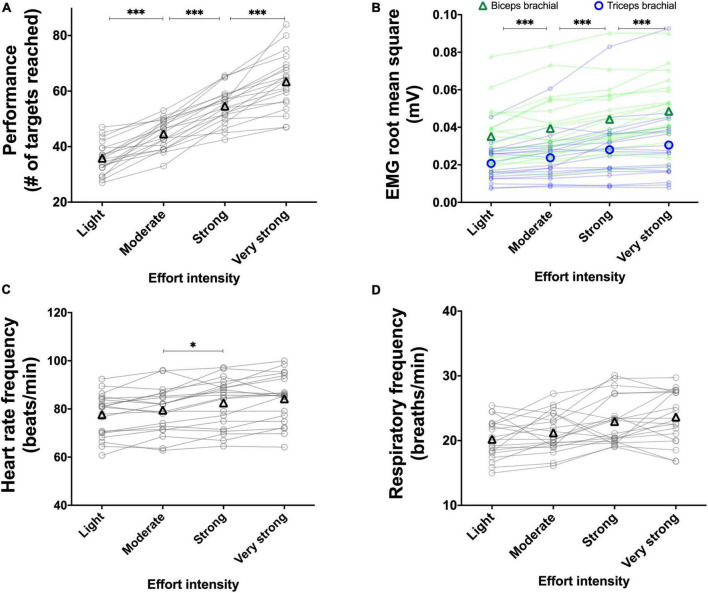
Experiment 1A: Using the perception of effort to prescribe the exercise during the pointing task. Effect of increasing the prescribed effort intensity on performance (**A**, *n* = 20), EMG root mean square of the biceps (green line) and triceps (blue line) brachial muscles (**B**, *n* = 20), heart rate frequency (**C**, *n* = 20), and respiratory frequency (**D**, *n* = 20) during the pointing task. The exercise was prescribed at four intensities of perceived effort *via* the CR100 scale: light (13/100), moderate (23/100), strong (50/100), and very strong (70/100). Data are presented as the main effect of effort intensity **(A, C, D)** and effort intensity × muscle interaction **(B)**. Individual data are presented in light markers and means in dark markers. *Main effect of intensity, the difference between two effort intensities. *p < 0.05, ***p < 0.001.

The results of the main effects of effort intensity for the BBT and PT are presented in Figures 3 and 4, respectively.

##### 3.1.1.1. Performance

For the BBT (Figure 3A), the main effect of visit did not reach significance [*F*(1, 19) = 2.105, *p* = 0.163, η_*p*_^2^ = 0.099]. Increasing the prescribed effort intensity resulted in an increased performance during the BBT [*F*(1.6, 31.2) = 172.335, *p <* 0.001, η_*p*_^2^ = 0.901]. The follow-up test revealed an increase in performance between the light and moderate intensities [*t*(19) = 10.509, *p* < 0.001, *r* = 0.924], between the moderate and strong intensities [*t*(19) = 10.474, *p* < 0.001, *r* = 0.923], as well as between the strong and very strong intensities [*t*(19) = 7.191, *p* < 0.001, *r* = 0.855]. The visit × effort intensity interaction did not reach significance [*F*(3, 57) = 0.401, *p* = 0.752, η_*p*_^2^ = 0.021]. For the PT (Figure 4A), the main effect of visit did not reach significance [*F*(1, 19) = 0.749, *p* = 0.397, η_*p*_^2^ = 0.038]. The main effect of effort intensity reached significance [*F*(1.6, 31.1) = 112.050, *p* < 0.001, η_*p*_^2^ = 0.855]. The follow-up test revealed an increase in performance between the light and moderate intensities [*t*(19) = 8.162, *p* < 0.001, *r* = 0.882], between the moderate and strong intensities [*t*(19) = 10.681, *p* < 0.001, *r* = 0.926], as well as between the strong and very strong intensities [*t*(19) = 6.291, *p* < 0.001, *r* = 0.822]. The visit × effort intensity interaction did not reach significance [*F*(1.4, 26.8) = 1.342, *p* = 0.270, η_*p*_^2^ = 0.065].

##### 3.1.1.2. RMS EMG

For the BBT (Figure 3B), the mean RMS EMG of the biceps brachii was higher than the mean RMS EMG of the triceps [*F*(1, 18) = 11.174, *p* = 0.003, η_*p*_^2^ = 0.081]. The main effect of visit did not reach significance [*F*(1, 18) = 2.018, *p* = 0.172, η_*p*_^2^ = 0.003]. There was a main effect of effort intensity [*F*(1.3, 24.7) = 37.667, *p* < 0.001, η_*p*_^2^ = 0.161] showing an increase between the light and moderate intensities [*t*(18) = 5.904, *p* < 0.001, *r* = 0.812], between the moderate and strong intensities [*t*(18) = 5.229, *p* < 0.001, *r* = 0.777], and between the strong and very strong intensities [*t*(18) = 4.109, *p* = 0.002, *r* = 0.696. The muscle × effort intensity interaction did not reach significance [*F*(1.6, 29.2) = 0.752, *p* = 0.454, η_*p*_^2^ = 0.001]. For the PT (Figure 4B), the mean RMS EMG of the biceps brachii was higher than the mean RMS EMG of the triceps [*F*(1, 19) = 14.477, *p* = 0.001, η_*p*_^2^ = 0.187]. The main effect of visit did not reach significance [*F*(1, 19) = 0.029, *p* = 0.866, η_*p*_^2^ < 0.001]. There was a main effect of effort intensity [*F*(1.2, 24.1) = 43.575, *p* < 0.001, η_*p*_^2^ = 0.085] showing an increase between the light and moderate intensities [*t*(19) = 6.410, *p* < 0.001, *r* = 0.827], between the moderate and strong intensities [*t*(19) = 5.541, *p* < 0.001, *r* = 0.786], and between the strong and very strong intensities [*t*(19) = 4.812, *p* < 0.001, *r* = 0.741]. The muscle × effort intensity interaction did not reach significance [*F*(1.3, 24.7) = 3.281, *p* = 0.072, η_*p*_^2^ = 0.002].

##### 3.1.1.3. Heart rate frequency

For the BBT (Figure 3C), the main effect of visit did not reach significance [*F*(1, 8) = 0.851, *p* = 0.383, η_*p*_^2^ = 0.096]. The main effect of effort intensity reached significance [*F*(3, 24) = 8.166, *p* = 0.001, η_*p*_^2^ = 0.505]. The follow-up tests revealed an increase in heart rate frequency between the moderate and strong intensities [*t*(17) = 3.176, *p* = 0.017, *r* = 0.610]. Neither the increase in heart rate frequency between the light and moderate intensities [*t*(17) = 1.490, *p* = 0.464, *r* = 0.340] nor the one between the strong and very strong intensities did reach significance [*t*(17) = 0.334, *p* = 1.000, *r* = 0.081]. The visit × effort intensity interaction did not reach significance [*F*(3, 24) = 0.896, *p* = 0.458, η_*p*_^2^ = 0.101]. For the PT (Figure 4C), the main effect of visit did not reach significance [*F*(1, 14) = 0.218, *p* = 0.647, η_*p*_^2^ = 0.015]. The main effect of effort reached significance [*F*(3, 42) = 14.804, *p* < 0.001, η_*p*_^2^ = 0.513]. The follow-up test revealed an increase in heart rate frequency between the moderate and strong intensities [*t*(19) = 3.285, *p* = 0.012, *r* = 0.602], but not between the light and moderate intensities [*t*(19) = 2.182, *p* = 0.126, *r* = 0.448] not between the strong and very strong intensities [*t*(19) = 1.941, *p* = 0.202, *r* = 0.407]. The visit × effort intensity interaction did not reach significance [*F*(3, 42) = 0.406, *p* = 0.748, η_*p*_^2^ = 0.028].

##### 3.1.1.4. Respiratory frequency

For the BBT (Figure 3D), the main effect of visit did not reach significance [*F*(1, 13) = 0.008, *p* = 0.930, η_*p*_^2^ = 0.001]. The main effect of effort intensity reached significance [*F*(3, 39) = 6.463, *p* = 0.001, η_*p*_^2^ = 0.332]. However, neither the increase in respiratory frequency between the light and moderate intensities [*t*(19) = 2.450, *p* = 0.072, *r* = 0.490], between the moderate and strong intensities [*t*(19) = 2.131, *p* = 0.139, *r* = 0.439], or between the strong and very strong intensities did reach significance [*t*(19) = 1.663, *p* = 0.338, *r* = 0.357]. The visit × effort intensity interaction did not reach significance [*F*(3, 39) = 0.084, *p* = 0.970, η_*p*_^2^ = 0.006]. For the PT (Figure 4D), the main effect of visit did not reach significance [*F*(1, 15) = 0.142, *p* = 0.711, η_*p*_^2^ = 0.009]. The main effect of effort intensity reached significance [*F*(3, 45) = 10.893, *p* < 0.001, η_*p*_^2^ = 0.421]. However, again, neither the increase in respiratory frequency between the light and moderate intensities [*t*(19) = 1.648, *p* = 0.347, *r* = 0.354], between the moderate and strong intensities [*t*(19) = 2.451, *p* = 0.072, *r* = 0.490], or between the strong and very strong intensities did reach significance [*t*(19) = 1.052, *p* = 0.917, *r* = 0.235]. The visit × effort intensity interaction did not reach significance [*F*(3, 45) = 0.195, *p* = 0.899, η_*p*_^2^ = 0.012].

#### 3.1.2. Experiment 1B: Manipulating the tempo to alter task difficulty

**FIGURE 5:**
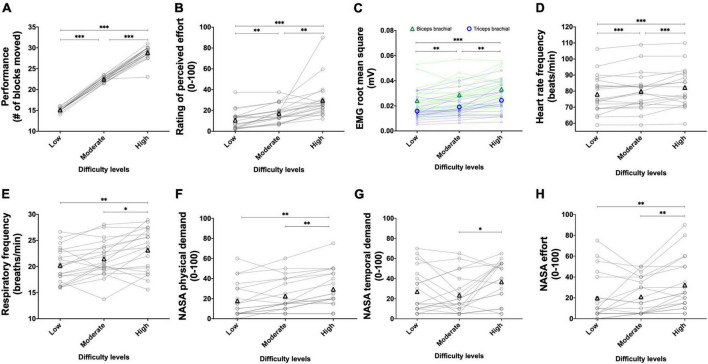
Experiment 1B: Manipulating the tempo to alter task difficulty during the box and block test. Effect of manipulating the tempo during the box and block test on performance (**A**, *n* = 20), rating of perceived effort (**B**, *n* = 20), EMG root mean square of the biceps (green line) and triceps (blue line) brachial muscles (**C**, *n* = 20), heart rate frequency (**D**, *n* = 18), respiratory frequency (**E**, *n* = 20) and NASA TLX scores for physical demand (**F**, *n* = 20), temporal demand (**G**, *n* = 20), and subjective effort (**H**, *n* = 20). For the low difficulty, a 0.5 Hz tempo was imposed. For moderate difficulty, a 0.75 Hz tempo was imposed. For the high difficulty, a 1 Hz tempo was imposed. Data are presented as the main effect of difficulty, except for panel **(C)** presenting the difficulty × muscle interaction. The n indicates the number of participants with all the data in each of the three levels of difficulties. Changes in the n reflect data loss due to issues with equipment or movement artifact. Individual data are presented in light markers and means in dark markers. *Main effect of difficulty, the difference between two difficulty levels. *p < 0.05, **p < 0.01, and ***p < 0.001.

**FIGURE 6:**
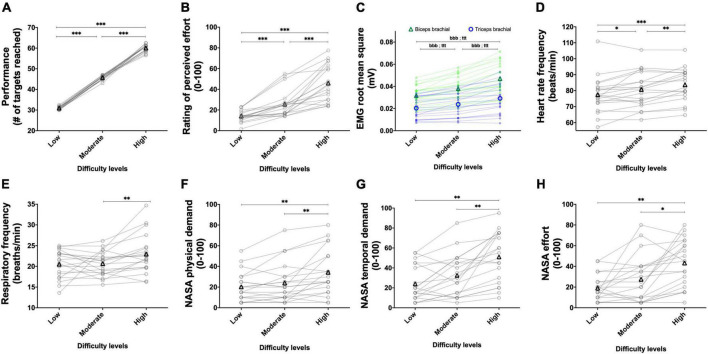
Experiment 1B: Manipulating the tempo to alter task difficulty during the pointing task. Effect of manipulating the tempo during the pointing task on performance (**A**, *n* = 20), rating of perceived effort (**B**, *n* = 20), EMG root mean square of the biceps (green line) and triceps (blue line) brachial muscles (**C**, *n* = 20), heart rate frequency (**D**, *n* = 18), respiratory frequency (**E**, *n* = 20) and NASA TLX scores for physical demand (**F**, *n* = 20), temporal demand (**G**, *n* = 20) and subjective effort (**H**, *n* = 20). For the low difficulty, a 1 Hz tempo was imposed. For the moderate difficulty, a 1.5 Hz tempo was imposed. For the high difficulty, a 2 Hz tempo was imposed. Data are presented as the main effect of difficulty, except for panel C presenting the difficulty × muscle interaction. The n indicates the number of participants with all the data in each of the three levels of difficulties. Changes in the n reflect data loss due to issues with equipment or movement artifact. Individual data are presented in light markers and means in dark markers. *Main effect of difficulty, the difference between two difficulty levels. *b* and *t* difference between two difficulty levels for the biceps and triceps brachial muscles, respectively. One symbol: *p* < 0.05, two symbols: *p* < 0.01, and three symbols: *p* < 0.001.

The results for the BBT and PT during the tempo sessions are presented in Figures 5 and 6, respectively.

##### 3.1.2.1. Performance

For the BBT (Figure 5A), manipulation of the tempo increased performance [χ^2^(2) = 40, *p* < 0.001]. Performance increased between the low and moderate difficulties (*Z* = 3.990, *p* < 0.001, *r* = 0.631), between the low and high difficulties (*Z* = 3.935, *p* < 0.001, *r* = 0.622), as well as between the moderate and high difficulties (*Z* = 3.941, *p* < 0.001, *r* = 0.623). One participant did not show an increase in performance between the moderate and high difficulties, as shown in the figure. For the PT (Figure 6A), manipulation of the tempo increased performance too [χ^2^(2) = 40, *p* < 0.001]. Performance increased between the low and moderate difficulties (*Z* = 3.965, *p* < 0.001, *r* = 0.627), between the low and high difficulties (*Z* = 3.941, *p* < 0.001, *r* = 0.623), as well as between the moderate and high difficulties (*Z* = 3.932, *p* < 0.001, *r* = 0.622).

##### 3.1.2.2. Perception of effort

For the BBT (Figure 5B), manipulation of the tempo increased the rating of perceived effort [χ^2^(2) = 30.152, *p* < 0.001]. Rating of perceived effort increased between the low and moderate difficulties (*Z* = 3.747, *p* = 0.001, *r* = 0.592), between the low and high difficulties (*Z* = 3.790, *p* < 0.001, *r* = 0.599), and between the moderate and high difficulties (*Z* = 3.460, *p* = 0.002, *r* = 0.547). For the PT (Figure 6B), manipulation of the tempo increased the rating of perceived effort too [χ^2^(2) = 36.1, *p* < 0.001]. Rating of perceived effort increased between the low and moderate difficulties (*Z* = 3.865, *p* < 0.001, *r* = 0.611), between the low and high difficulties (*Z* = 3.921, *p* < 0.001, *r* = 0.620), as well as between the moderate and high difficulties (*Z* = 3.883, *p* < 0.001, *r* = 0.614).

##### 3.1.2.3. RMS EMG

For the BBT (Figure 5C), the mean RMS EMG of the biceps brachii was higher than the mean RMS EMG of the triceps [*F*(1, 19) = 10.441, *p* = 0.004, η_*p*_^2^ = 0.355]. There was a main effect of difficulty [*F*(1.46, 27.73) = 22.851, *p* < 0.001, η_*p*_^2^ = 0.546], showing an increase between the low and moderate difficulties [*t*(19) = 4.29, *p* = 0.001, *r* = 0.701], the low and high difficulties [*t*(19) = 5.44, *p* < 0.001, *r* = 0.780], and the moderate and high difficulties [*t*(19) = 3.81, *p* = 0.004, *r* = 0.658]. The difficulty × muscle interaction did not reach significance [*F*(2, 38) = 0.376, *p* = 0.689, η_*p*_^2^ = 0.019]. For the PT (Figure 6C), the mean RMS EMG of the biceps brachii was higher than the mean RMS EMG of the triceps [*F*(1, 19) = 15.95, *p* < 0.001, η_*p*_^2^ = 0.456]. There was a main effect of difficulty [*F*(1.21, 22.95) = 132.51, *p* < 0.001, η_*p*_^2^ = 0.875], showing an increase between the low and moderate difficulties [*t*(19) = 9.43, *p* < 0.001, *r* = 0.908], the low and high difficulties [*t*(19) = 12.07, *p* < 0.001, *r* = 0.941], and the moderate and high difficulties [*t*(19) = 11.33, *p* < 0.001, *r* = 0.933]. The difficulty × muscle interaction reached significance [*F*(1.28, 24.31) = 7.26, *p* = 0.008, η_*p*_^2^ = 0.276]. Follow-up tests are presented in Figure 6C.

##### 3.1.2.4. Heart rate frequency

Despite controlling for movement artifacts, data were lost in two participants during the BBT and two participants during the PT, both during the completion of the high difficulty. For the BBT (Figure 5D), manipulation of the tempo increased the heart rate frequency [*F*(2, 34) = 9.826, *p* < 0.001, η_*p*_^2^ = 0.366]. Heart rate frequency increased between the low and moderate difficulties [*t*(19) = 2.517, *p* < 0.001, *r* = 0.500], between the low and high difficulties [*t*(17) = 3.861, *p* < 0.001, *r* = 0.684], as well as between the moderate and high difficulties [*t*(17) = 2.297, *p* < 0.001, *r* = 0.487]. For the PT (Figure 6D), manipulation of the tempo increased the heart rate frequency too [*F*(2, 34) = 15.707, *p* < 0.001, η_*p*_^2^ = 0.480]. Heart rate frequency increased between the low and moderate difficulties [*t*(19) = 2.707, *p* = 0.042, *r* = 0.528], between the low and high difficulties [*t*(17) = 4.911, *p* < 0.001, *r* = 0.766], and between the moderate and high difficulties [*t*(17) = 3.604, *p* = 0.007, *r* = 0.658].

##### 3.1.2.5. Respiratory frequency

For the BBT (Figure 5E), manipulation of the tempo increased the respiratory frequency [*F*(2, 38) = 10.5, *p* < 0.001, η_*p*_^2^ = 0.355]. The increase in respiratory frequency between the low and moderate difficulties did not reach significance [*t*(19) = 2.373, *p* = 0.085, *r* = 0.478]. Respiratory frequency increased between the low and high difficulties [*t*(19) = 3.797, *p* = 0.004, *r* = 0.657] as well as between the moderate and high difficulties [*t*(19) = 2.8, *p* = 0.036, *r* = 0.537]. For the PT (Figure 6E), manipulation of the tempo increased the respiratory frequency too [*F*(2, 38) = *5.3, p* = 0.009, η_*p*_^2^ = 0.219]. Respiratory frequency increased between the moderate and high difficulties [*t*(19) = 3.380, *p* = 0.009, *r* = 0.613]. The increase in respiratory frequency neither reached significance between the low and high difficulties [*t*(19) = 2.391, *p* = 0.082, *r* = 0.481] nor between the low and moderate difficulties [*t*(19) = 0.184, *p* = 1.000, *r* = 0.042].

##### 3.1.2.6. NASA TLX scale, physical demand

For the BBT (Figure 5F), manipulation of the tempo increased the physical demand score [χ^2^(2) = 17.815, *p* < 0.001]. The increase in physical demand score between the easy and medium difficulties did not reach significance (*Z* = 2.213, *p* = 0.081, *r* = 0.350). The physical demand score increased between the low and high difficulties (*Z* = 3.307, *p* = 0.003, *r* = 0.523) as well as between the moderate and high difficulties (*Z* = 3.051, *p* = 0.007, *r* = 0.482). For the PT (Figure 6F), manipulation of the tempo increased the physical demand score too [χ^2^(2) = 14.464, *p* = 0.001]. The physical demand score did not increase between the low and moderate difficulties (*Z* = 1.690, *p* = 0.273, *r* = 0.267). The physical demand score increased between the low and high difficulties (*Z* = 3.354, *p* = 0.002, *r* = 0.530) as well as between the moderate and high difficulties (*Z* = 3.066, *p* = 0.007, *r* = 0.485).

##### 3.1.2.7. NASA TLX scale, mental demand

For the BBT, manipulation of the tempo increased the mental demand score [χ^2^(2) = 15.672, *p* < 0.001]. The increase in mental demand score between the low (19.5 ± 17.2 a.u.) and moderate (24.3 ± 16.2 a.u.) difficulties did not reach significance (*Z* = 1.825, *p* = 0.204, *r* = 0.289). Mental demand score increased between the low and high (35.3 ± 23.3 a.u.) difficulties (*Z* = 3.196, *p* = 0.004, *r* = 0.505) and between the moderate and high difficulties (*Z* = 3.219, *p* = 0.004, *r* = 0.509). For the PT, manipulation of the tempo increased the mental demand score [χ^2^(2) = 12.649, *p* = 0.002]. The increase in mental demand score between the low (22.3 ± 12.4 a.u.) and moderate (30.5 ± 21.0 a.u.) difficulties did not reach significance (*Z* = 1.556, *p* = 0.359, *r* = 0.246). The mental demand score increased between the low and high (40.8 ± 22.6 a.u.) difficulties (*Z* = 3.012, *p* = 0.008, *r* = 0.476) and between the moderate and high difficulties (*Z* = 2.710, *p* = 0.020, *r* = 0.428).

##### 3.1.2.8. NASA TLX scale, temporal demand

For the BBT (Figure 5G), manipulation of the tempo increased the temporal demand score [χ^2^(2) = 7.28, *p* = 0.026]. The temporal demand score neither increased between the low and moderate difficulties (*Z* = 0.572, *p* = 1.000, *r* = 0.090) nor between the low and high difficulties (*Z* = 2.194, *p* = 0.085, *r* = 0.347). The temporal demand score significantly increased between the moderate and high difficulties (*Z* = 2.686, *p* = 0.022, *r* = 0.425). For the PT (Figure 6G), manipulation of the tempo increased the temporal demand score too [χ^2^(2) = 23.792, *p* < 0.001]. The increase in temporal demand score between the low and moderate difficulties did not reach significance (*Z* = 2.144, *p* = 0.096, *r* = 0.339). The temporal demand score significantly increased between the low and high difficulties (*Z* = 3.712, *p* = 0.001, *r* = 0.587) as well as between the moderate and high difficulties (*Z* = 3.736, *p* = 0.001, *r* = 0.591).

##### 3.1.2.9. NASA TLX scale, effort

For the BBT (Figure 5H), manipulation of the tempo increased the effort score [χ^2^(2) = 18.123, *p* < 0.001]. Effort score did not increase between the low and moderate difficulties (*Z* = 0.177, *p* = 1.000, *r* = 0.028) but did so between the low and high difficulties (*Z* = 3.184, *p* = 0.004, *r* = 0.503), as well as between the moderate and high difficulties (*Z* = 3.202, *p* = 0.004, *r* = 0.506). For the PT (Figure 6H), manipulation of the tempo increased the effort demand score too [χ^2^(2) = 22.776, *p* < 0.001]. Effort score did not increase between the low and moderate difficulties (*Z* = 1.759, *p* = 0.236, *r* = 0.278) but did so between the low and high difficulties (*Z* = 3.637, *p* = 0.001, *r* = 0.575), as well as between the moderate and high difficulties (*Z* = 2.882, *p* = 0.012, *r* = 0.456).

##### 3.1.2.10. VAS fatigue

Feelings of fatigue did not increase during the tempo session (from 2.9 ± 2.2 to 3.2 ± 1.9; *Z* = 0.952, *p* = 0.340).

#### 3.1.3. Experiment 1B: Adding weight on the forearm to alter task difficulty

**FIGURE 7:**
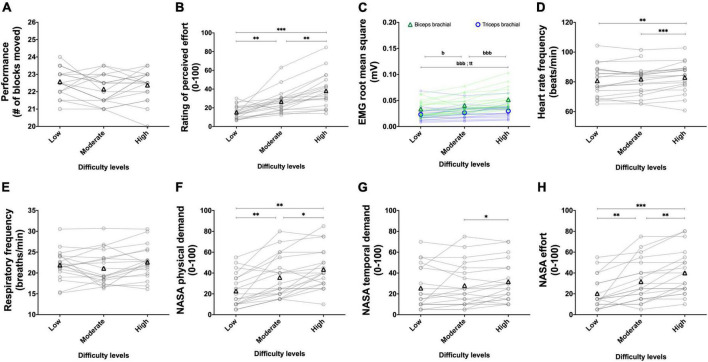
Experiment 1B: Adding weight on the forearm to alter task difficulty during the box and block test. The effect of manipulating the weight during the box and block test on performance (**A**, *n* = 20), rating of perceived effort (**B**, *n* = 20), EMG root mean square of the biceps (green line) and triceps (blue line) brachial muscles (**C**, *n* = 20), heart rate frequency (**D**, *n* = 16), respiratory frequency (**E**, *n* = 20) and NASA TLX scores for the physical demand (**F**, *n* = 20), the temporal demand (**G**, *n* = 20), and the subjective effort (**H**, *n* = 20). Movements were performed at a fixed tempo of 0.75 Hz. For the low difficulty, no additional weight on the forearm was added. For the moderate difficulty, a weight of 0.5 kg was added. For the high difficulty, a weight of 1 kg was added. Data are presented as the main effect of difficulty, except for panel **(C)** presenting the difficulty × muscle interaction. The n indicates the number of participants with all the data in each of the three levels of difficulties. Changes in the n reflect data loss due to issues with equipment or movement artifact. Individual data are presented in light markers and means in dark markers. *Main effect of difficulty, the difference between two difficulty levels. *b* and *t* difference between two difficulty levels for the biceps and triceps brachial muscles, respectively. One symbol: *p* < 0.05, two symbols: *p* < 0.01, and three symbols: *p* < 0.001.

**FIGURE 8:**
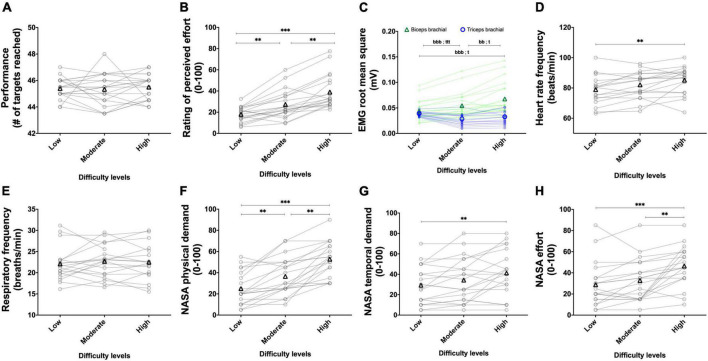
Experiment 1B: Adding weight on the forearm to alter task difficulty during the pointing task. Effect of manipulating the weight during the pointing Task on performance (**A**, *n* = 20), rating of perceived effort (**B**, *n* = 20), EMG root mean square of the biceps (green line) and triceps (blue line) brachial muscles (**C**, *n* = 20), heart rate frequency (**D**, *n* = 17), respiratory frequency (**E**, *n* = 20) and NASA TLX scores for the physical demand (**F**, *n* = 20), the temporal demand (**G**, *n* = 20) and the subjective effort (**H**, *n* = 20). Movements were performed at a fixed tempo of 1.5 Hz. For the low difficulty, no additional weight on the forearm was added. For the moderate difficulty, a weight of 0.5 kg was added. For the high difficulty, a weight of 1 kg was added. Data are presented as the main effect of difficulty, except for panel **(C)** presenting the difficulty × muscle interaction. The n indicates the number of participants with all the data in each of the three levels of difficulties. Changes in the n reflect data loss due to the equipment. Individual data are presented in gray circles and means in black triangles. *Main effect of difficulty, the difference between two difficulty levels. *b* and *t* difference between two difficulty levels for the biceps and triceps brachial muscles, respectively. One symbol: *p* < 0.05, two symbols: *p* < 0.01, and three symbols: *p* < 0.001.

The results for the BBT and PT during the weight sessions are presented in Figures 7 and 8, respectively.

##### 3.1.3.1. Performance

For the BBT (Figure 7A) and PT (Figure 8A), manipulation of the weight did not alter performance [BBT, χ^2^(2) = 4.899, *p* = 0.086; PT, χ^2^(2) = 2.032, *p* = 0.362].

##### 3.1.3.2. Perception of effort

For the BBT (Figure 7B), manipulation of the weight increased the rating of perceived effort [χ^2^(2) = 36.026, *p* < 0.001]. Rating of perceived effort increased between the low and moderate difficulties (*Z* = 3.341, *p* = 0.003, *r* = 0.528), between the low and high difficulties (*Z* = 3.921, *p* < 0.001, *r* = 0.620), and between the moderate and high difficulties (*Z* = 3.624 *p* = 0.001, *r* = 0.573). For the PT (Figure 8B), manipulation of the weight increased the rating of perceived effort too [χ^2^(2). = 32.076, *p* < 0.001]. Rating of perceived effort increased between the low and moderate difficulties (*Z* = 3.324, *p* = 0.003, *r* = 0.526), between the low and high difficulties (*Z* = 3.920, *p* < 0.001, *r* = 0.620), and between the moderate and high difficulties (*Z* = 3.502, *p* = 0.001, *r* = 0.554).

##### 3.1.3.3. RMS EMG

For the BBT (Figure 7C), the mean RMS EMG of the biceps brachii was higher than the mean RMS EMG of the triceps [*F*(1, 19) = 11.339, *p* = 0.003, η_*p*_^2^ = 0.374]. There was a main effect of difficulty [*F*(1.27, 24.08) = 25.276, *p* < 0.001, η_*p*_^2^ = 0.571] showing an increase between the low and moderate difficulties [*t*(19) = 2.954, *p* = 0.024, *r* = 0.561], between the moderate and high difficulties [*t*(19) = 7.065, *p* < 0.001, *r* = 0.851] as well as between the low and high difficulties [*t*(19) = 5.499, *p* < 0.001, *r* = 0.784]. The difficulty × muscle interaction reached significance [*F*(2, 38) = 14.857, *p* < 0.001, η_*p*_^2^ = 0.438]. Follow-up tests are presented in Figure 7C for the PT (Figure 8C), the mean RMS EMG of the biceps brachii was higher than the mean RMS EMG of the triceps [*F*(1, 19) = 11.001, *p* = 0.003, η_*p*_^2^ = 0.367]. There was a main effect of difficulty [*F*(1.33, 25.20) = 13.148, *p* < 0.001, η_*p*_^2^ = 0.409] showing an increase and between the moderate and high difficulties [*t*(19) = 3.974, *p* < 0.01, *r* = 0.674] and between the low and high difficulties [*t*(19) = 3.686, *p* < 0.01, *r* = 0.646], but not between the low and moderate difficulties [*t*(19) = 0.048, *p* > 0.05, *r* = 0.011]. The difficulty × muscle interaction reached significance [*F*(1.30, 24.74) = 48.057, *p* < 0.001, η_*p*_^2^ = 0.717]. Follow-up tests are presented in Figure 8C.

##### 3.1.3.4. Heart rate frequency

Despite controlling for movement artifacts, data were lost during the BBT in four participants during the completion of the moderate difficulty and in one participant during the completion of the high difficulty. During the PT, data were lost in two participants during the completion of the low difficulty, in one participant during the completion of the moderate difficulty and in one participant during the completion of the high difficulty. For the BBT (Figure 7D), manipulation of the weight increased the heart rate frequency [*F*(2, 30) = 13.758, *p* < 0.001, η_*p*_^2^ = 0.478]. Heart RM rate frequency did not increase between the low and moderate difficulties [*t*(15) = 0.748, *p* = 1.000, *r* = 0.190] but did so between the low and high difficulties [*t*(15) = 4.213, *p* = 0.002, *r* = 0.736], as well as between the moderate and high difficulties [*t*(15) = 5.115, *p* < 0.001, *r* = 0.797]. For the PT (Figure 8D), manipulation of the weight significantly increased the heart rate frequency too [*F*(2, 32) = 11.257, *p* < 0.001, η_*p*_^2^ = 0.413]. The increase in the heart rate frequency between the low and moderate difficulties [*t*(16) = 2.636, *p* = 0.054, *r* = 0.550] as well as between the moderate and high difficulties [*t*(16) = 2.541, *p* = 0.065, *r* = 0.536] did not reach significance. Heart rate frequency significantly increased between the low and high difficulties [*t*(16) = 4.190, *p* = 0.002, *r* = 0.723].

##### 3.1.3.5. Respiratory frequency

During the BBT, data were lost in one participant during the completion of both the low and high difficulties. During the PT, data were lost in one participant for the three difficulties and in one participant during the high difficulty. For the BBT (Figure 7E) and PT (Figure 8E), manipulation of the weight did not alter respiratory frequency [BBT, *F*(2, 36) = 1.931, *p* = 0.159, η_*p*_^2^ = 0.097; PT, *F*(2, 34) = 1.477, *p* = 0.243, η_*p*_^2^ = 0.080].

##### 3.1.3.6. NASA TLX scale, and physical demand

For the BBT (Figure 7F), manipulation of the weight increased the physical demand score [χ^2^(2) = 18.2, *p* < 0.001]. Physical demand score increased between the low and moderate difficulties (*Z* = 3.373, *p* = 0.002, *r* = 0.533), between the moderate and high difficulties (*Z* = 2.630, *p* = 0.026, *r* = 0.416), and between the low and high difficulties (*Z* = 3.497, *p* = 0.001, *r* = 0.553). For the PT (Figure 8F), manipulation of the weight significantly increased the physical demand score too [χ^2^(2) = 35.351, *p* < 0.001]. Physical demand score increased between the low and moderate difficulties (*Z* = 3.218, *p* = 0.004, *r* = 0.509), between the moderate and high difficulties (*Z* = 3.734 *p* = 0.001, *r* = 0.590), and between the low and high difficulties (*Z* = 3.930, *p* < 0.001, *r* = 0.621).

##### 3.1.3.7. NASA TLX scale, mental demand

For the BBT, manipulation of the tempo increased the mental demand score [χ^2^(2) = 8.400, *p* = 0.015]. The mental demand score increased between the low (22.5 ± 15.6 a.u.) and moderate (29.3 ± 17.6 a.u.) difficulties (*Z* = 2.695, *p* = 0.021, *r* = 0.426) as well as between the low and high (29.8 ± 19.3 a.u.) difficulties (*Z* = 2.435, *p* = 0.045, *r* = 0.385). The mental demand score did not increase between the moderate and high difficulties (*Z* = 0.109, *p* = 1.000, *r* = 0.017). For the PT, manipulation of the tempo increased the mental demand score [χ^2^(2) = 7.750, *p* = 0.021]. The increase in the mental demand score between the low (27.3 ± 14.0 a.u.) and moderate (36.5 ± 21.5 a.u.) difficulties did not reach significance (*Z* = 2.226, *p* = 0.078, *r* = 0.352). The mental demand score increased between the low and high (42.5 ± 17.9 a.u.) difficulties (*Z* = 3.274, *p* = 0.003, *r* = 0.518). The mental demand score did not increase between the moderate and high difficulties (*Z* = 1.706, *p* = 0.264, *r* = 0.270).

##### 3.1.3.8. NASA TLX scale, temporal demand

For the BBT (Figure 7G), manipulation of the weight increased the temporal demand score [χ^2^(2). = 7, *p* = 0.031]. The temporal demand score did not increase between the low and moderate difficulties (*Z* = 0.361, *p* = 1.000, *r* = 0.057), as well as between the low and high difficulty (*Z* = 1.934, *p* = 0.159, *r* = 0.306), but increased between the moderate and high difficulty (*Z* = 2.423, *p* = 0.046, *r* = 0.383). For the PT (Figure 8G), manipulation of the weight increased the temporal demand score too [χ^2^(2) = 8.222, *p* = 0.016]. The temporal demand score did not increase between the low and moderate difficulties (*Z* = 2.042, *p* = 0.123, *r* = 0.323), as well as between the moderate and high difficulties (*Z* = 2.110, *p* = 0.105, *r* = 0.334), but increased between the low and high difficulties (*Z* = 3.086, *p* = 0.006, *r* = 0.488).

##### 3.1.3.9. NASA TLX scale, effort

For the BBT (Figure 7H), manipulation of the weight increased the effort score [χ^2^(2) = 28.353, *p* < 0.001]. The effort score increased between the low and moderate difficulties (*Z* = 3.309, *p* = 0.003, *r* = 0.523), between the moderate and high difficulties (*Z* = 3.225, *p* = 0.004, *r* = 0.510), as well as between the low and high difficulties (*Z* = 3.798, *p* < 0.001, *r* = 0.601). For the PT (Figure 8H), manipulation of the weight increased the effort score [χ^2^(2) = 25.507, *p* < 0.001]. The effort score did not increase between the low and moderate difficulties (*Z* = 1.720, *p* = 0.256, *r* = 0.272), but did so between the moderate and high difficulties (*Z* = 3.362, *p* = 0.002, *r* = 0.532), as well as between the low and high difficulties (*Z* = 3.604, *p* = 0.001, *r* = 0.570).

##### 3.1.3.10. VAS fatigue

Feelings of fatigue increased during the tempo session (from 3.1 ± 2.3 to 3.9 ± 1.9; *Z* = 2.315, *p* = 0.021).

### 3.2. Experiment 2

In this experiment, participants visited the laboratory once. In Experiment 2A, we prescribed 30 s of exercise with the BBT performed at four intensities of effort (light, moderate, strong, and very strong). Performance, RMS EMG, and heart rate frequency were monitored for each prescribed effort intensity. Then, in Experiment 2B, we manipulated task difficulty (low, high) by adding two different weights on the participant’s dominant forearm while performing the standardized 60 s BBT. Each level of difficulty was repeated twice. Performance, rating of perceived effort, RMS EMG heart rate frequency, and the subjective workload were measured for each repetition of each level of difficulty.

#### 3.2.1. Experiment 2A: Using the perception of effort to prescribe the exercise

**FIGURE 9:**
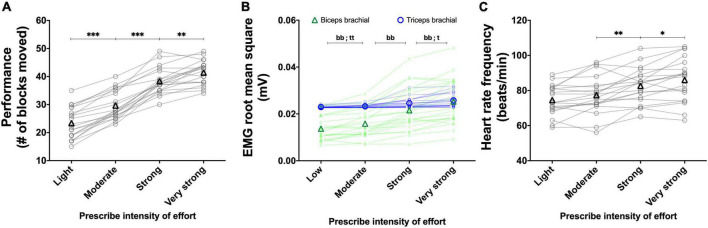
Experiment 2A: Using the perception of effort to prescribe the exercise during the box and block test. Effect of increasing the prescribed intensity of effort on performance (**A**, *n* = 20), EMG root mean square of the biceps (green line) and triceps (blue line) brachial muscles (**B**, *n* = 20), heart rate frequency (**C**, *n* = 20) during the box and block test. The exercise was prescribed at four intensities of perceived effort *via* the CR100 scale: light (13/100), moderate (23/100), strong (50/100), and very strong (70/100). Data are presented as the main effect of effort intensity, except for panel **(B)** presenting the effort intensity × muscle interaction. Individual data are presented in light markers and means in dark markers. *Main effect of difficulty, the difference between two difficulty levels. *b* and *t* are the difference between two difficulty levels for the biceps and triceps brachial muscles, respectively. One symbol: *p* < 0.05, two symbols: *p* < 0.01, and three symbols: *p* < 0.001.

Results are presented in Figure 9.

##### 3.2.1.1. Performance

Increasing the prescribed effort intensity resulted in an increased performance [*F*(1.7, 31.6) = 168.560, *p* < 0.001, η_*p*_^2^ = 0.899; Figure 9A]. Performance increased between the light and moderate effort intensities [*t*(19) = 11.393, *p* < 0.001, *r* = 0.934], between moderate and strong effort intensities [*t*(19) = 12.564, *p* < 0.001, *r* = 0.945], and between strong and very strong effort intensities [*t*(19) = 4.258, *p* = 0.001, *r* = 0.699].

##### 3.2.1.2. RMS EMG

Mean RMS EMG of the biceps brachii was lower than the mean RMS EMG of the triceps [*F*(1, 19) = 11.285, *p* = 0.003, η_*p*_^2^ = 0.373]. There was a main effect of effort intensity [*F*(1.41, 26.76) = 36.852, *p* < 0.001, η_*p*_^2^ = 0.659], showing an increase between the light and moderate intensities [*t*(19) = 4.471, *p* < 0.001, *r* = 0.716], between the moderate and strong intensities [*t*(19) = 5.235, *p* < 0.001, *r* = 0.769], and between the strong and very strong [*t*(19) = 4.310, *p* = 0.001, *r* = 0.703].]. The muscle x effort intensity interaction reached significance [*F*(1.45, 27.56) = 38.540, *p* < 0.001, η_*p*_^2^ = 0.670]. Follow-up tests are presented in Figure 9B.

##### 3.2.1.3. Heart rate frequency

Increasing the prescribed effort intensity resulted in an increased heart rate [*F*(3, 57) = 29.074, *p* < 0.001, η_*p*_^2^ = 0.605; Figure 9C]. The increase in heart rate frequency between the light and moderate effort intensities did not reach significance [*t*(19) = 2.316, *p* = 0.096, *r* = 0.469]. Heart rate frequency significantly increased between the moderate and strong difficulty [*t*(19) = 4.027, *p* = 0.002, *r* = 0.679], and between strong and very strong effort intensities [*t*(19) = 2.925, *p* = 0.026, *r* = 0.557].

#### 3.2.2. Experiment 2B: Effects of adding weight on the forearm when completing the box and block test with the standardized instructions

**FIGURE 10:**
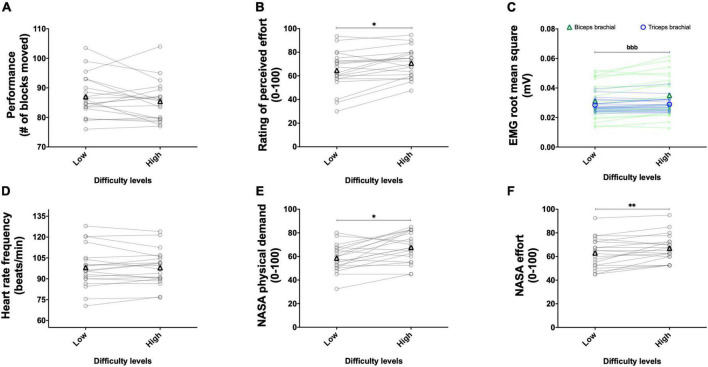
Experiment 2B: Adding weight on the forearm to alter task difficulty during the box and block test with its validated instructions. Effect of weight manipulation on performance (**A**, *n* = 20), rating of perceived effort (**B**, *n* = 20), EMG root mean square of the biceps (green line) and triceps (blue line), brachial muscles (**C**, *n* = 20), heart rate frequency (**D**, *n* = 20), and NASA TLX scores for physical demand (**E**, *n* = 20) and effort (**F**, *n* = 20) during the box and block test with its official instructions. Data are presented as the main effect of difficulty, except for panel **(C)** presenting the effort difficulty × muscle interaction. Individual data are presented in light markers and means in dark markers. *Main effect of difficulty, the difference between two difficulty levels. *b* is the difference between two difficulty levels for the biceps and triceps brachial muscles, respectively. One symbol: *p* < 0.05 and two symbols: *p* < 0.01.

The results of the main effects of difficulty are presented in Figure 10.

##### 3.2.2.1. Performance

The main effect of repetition revealed a greater performance in the second repetition compared to the first repetition [*F*(1, 19) = 34.836, *p* < 0.001, η_*p*_^2^ = 0.647]. The main effect of difficulty did not reach significance [*F*(1, 19) = 1.867, *p* = 0.188, η_*p*_^2^ = 0.090; Figure 10A]. The repetition × difficulty interaction reached significance [*F*(1, 19) = 5.166, *p* = 0.035, η_*p*_^2^ = 0.214]. Follow-up tests revealed an increased performance between the first and second repetitions for both the low {from 84.3 ± 6.6 to 89.7 ± 8.0; [*t*(19) = 5.219, *p* < 0.001, *r* = 0.768]} and high {from 84.0 ± 7.0 to 86.8 ± 6.9; [*t*(19) = 3.667, *p* = 0.005, *r* = 0.644]} difficulties. Performance did not differ for the first repetition between the low and high difficulties {84.3 ± 6.6 and 84.0 ± 7.0; [*t*(19) = 0.188, *p* = 1.000, *r* = 0.043]}. During the second repetition, performance did not significantly decrease between the low and high difficulties {89.7 ± 8.0 and 86.8 ± 6.9; [*t*(19) = 2.316, *p* = 0.096, *r* = 0.469]}.

##### 3.2.2.2. Perception of effort

The main effect of repetition revealed a higher rating of perceived effort in the second repetition compared to the first repetition [*F*(1, 19) = 14.350, *p* = 0.001, η_*p*_^2^ = 0.430]. The main effect of difficulty revealed an increase in the rating of perceived effort with the increase in difficulty [*F*(1, 19) = 6.779, *p* = 0.017, η_*p*_^2^ = 0.263; Figure 10B]. The repetition × difficulty interaction did not reach significance [*F*(1, 19) = 0.005, *p* = 0.946, η_*p*_^2^ < 0.001].

##### 3.2.2.3. RMS EMG

The main effect of muscle did not reach significance [*F*(1, 19) = 3.024, *p* = 0.098, η_*p*_^2^ = 0.137]. The main effect of repetition revealed a higher RMS EMG in the second repetition compared to the first repetition [*F*(1, 19) = 11.677, *p* = 0.003, η_*p*_^2^ = 0.381]. The main effect of difficulty revealed an increase in RMS EMG with the increase in difficulty [*F*(1, 19) = 14.289, *p* = 0.001, η_*p*_^2^ = 0.429]. The muscle × difficulty interaction reached significance [*F*(1, 19) = 20.525, *p* < 0.001, η_*p*_^2^ = 0.519], follow-up tests are presented in Figure 10C. The muscle × repetition interaction [*F*(1, 19) = 0.378, *p* = 0.546, η_*p*_^2^ = 0.019], difficulty × repetition interaction [*F*(1, 19) < 0.001, *p* = 0.978, η_*p*_^2^ < 0.001], and muscle × difficulty × repetition interaction [*F*(1, 19) = 0.032, *p* = 0.860, η_*p*_^2^ = 0.002] did not reach significance.

##### 3.2.2.4. Heart rate frequency

Main effect of repetition [*F*(1, 19) = 1.094, *p* = 0.309, η_*p*_^2^ = 0.054], difficulty [*F*(1, 19) = 0.664, *p* = 0.425, η_*p*_^2^ = 0.034; Figure 10D], and repetition × difficulty interaction [*F*(1, 19) = 0.492, *p* = 0.492, η_*p*_^2^ = 0.025] did not reach significance.

##### 3.2.2.5. NASA TLX scale, physical demand

The main effect of repetition revealed a higher physical demand score in the second repetition compared to the first repetition [*F*(1, 19) = 20.328, *p* < 0.001, η_*p*_^2^ = 0.517]. The main effect of difficulty revealed an increase in physical demand score with the increase in difficulty [*F*(1, 19) = 13.426, *p* = 0.002, η_*p*_^2^ = 0.414; Figure 10E]. The repetition × difficulty interaction did not reach significance [*F*(1, 19) = 1.342, *p* = 0.261, η_*p*_^2^ = 0.066].

##### 3.2.2.6. NASA TLX scale, mental demand

The main effect of repetition revealed a higher mental demand score in the second (49.4 ± 28.4 a.u.) repetition compared to the first (44.8 ± 25.8 a.u.) repetition [*F*(1, 19) = 4.916, *p* = 0.039, η_*p*_^2^ = 0.206]. Neither the main effect of difficulty [*F*(1, 19) = 0.514, *p* = 0.482, η_*p*_^2^ = 0.026] nor the difficulty × repetition interaction [*F*(1, 19) = 0.112, *p* = 0.742, η_*p*_^2^ = 0.006] reached significance.

##### 3.2.2.7. NASA TLX scale, effort

The main effect of repetition did not reach significance [*F*(1, 19) = 2.664, *p* = 0.119, η_*p*_^2^ = 0.123]. The main effect of difficulty revealed an increase in effort score with the increase in difficulty [*F*(1, 19) = 8.780, *p* = 0.008, η_*p*_^2^ = 0.316; Figure 10F]. The repetition × difficulty interaction did not reach significance [*F*(1, 19) = 0.039, *p* = 0.846, η_*p*_^2^ = 0.002].

##### 3.2.2.8. VAS fatigue

Feelings of fatigue did not increase during the session (from 5.75 ± 0.6 to 5 ± 1.7; *Z* = 1.916, *p* = 0.055).

## 4. Discussion

In this study, we investigated the possibility to prescribe and monitor exercise with the perception of effort during two upper-limb motor tasks: the box and block test and a pointing task. Our results suggest that performance in both tasks increased when the perception of effort intensity used to prescribe the exercise increased. When the task difficulty was altered by manipulating the physical demand *via* different tempos or weights added on the forearm, our results suggest that perception of effort increased when task difficulty increased and that performance could be maintained at a cost of a higher perception of effort. This increased perception of effort was observed during both the modified version of the box and block test as well as the pointing task performed in experiment 1. Finally, when completing the standardized version of the box and block test in the absence and presence of additional weight on the forearm, in experiment 2, we observed a maintained performance at a cost of a higher perception of effort. Overall, the results from both experiments suggest that perception of effort can be efficiently used in healthy young adults to prescribe and monitor physical resources allocation during upper-limb motor tasks.

### 4.1. Perception of effort can be used to prescribe the exercise intensity of upper-limb motor tasks

Perception of effort is widely used in the field of exercise sciences to prescribe exercise (Borg, 1998; Eston and Parfitt, 2018). As an example, the intensity of perception of effort has been used to prescribe locomotor exercise such as running or cycling (e.g., Christian et al., 2014; Hobbins et al., 2019), and resistance exercise involving the upper and lower limb (e.g., Gearhart et al., 2009; Zourdos et al., 2016; Helms et al., 2017). However, to the best of our knowledge, the possibility to use the intensity of perception of effort for exercise prescription in the context of upper-limb motor tasks remains untested. As the intensity of effort engaged in a task is proposed to determine the performance in this task (Brehm and Self, 1989; Richter et al., 2016), performance should increase when the intensity of perceived effort increases. We tested this possibility in both experiments. In experiment 1, we observed, during the box and block test and a pointing task, a gradual increase in performance between each intensity of perceived effort used to prescribe the exercise. This observation was subsequently reproduced in experiment 2 with another sample of participants performing the regular box and block test. Therefore, as previously observed during locomotor exercise or resistance exercise, our results suggest that the intensity of perceived effort could be an efficient tool to prescribe the exercise during upper-limb motor tasks. Interestingly, we did not observe any main effect of visit on performance for prescribing exercise during upper-limb motor tasks. This result suggests that our familiarization with the CR100 scale and associated instructions, combined with a 1-min practice of the tasks, was sufficient to control for a familiarization effect. In other words, when using the CR100 scale and associated instructions, our results imply that it is not necessary to perform an extensive practice of the motor tasks (e.g., exploring all range of intensity) to use the CR100 in the context of exercise prescription. This result is of great interest for researchers and clinicians willing to explore the use of this scale as it suggests that its use could be time-efficient when an extensive familiarization with the motor task is not possible due to time constraints.

To further confirm the possibility to use the perception of effort to prescribe exercise, we also monitored several physiological responses to the task performed: muscle activation, heart rate, and respiratory frequencies. These physiological responses are known to rise when the intensity of a task is increased during locomotor exercise as well as resistance exercise (de Morree and Marcora, 2010, 2012; Eston and Parfitt, 2018); we, therefore, hypothesized that the physiological responses would rise with the increased perceived effort intensity. As expected, all physiological parameters rose with the increased exercise intensity, confirming an increase in physical resources involved in the upper-limb motor tasks performed when the prescribed perceived effort intensity increased. However, it is crucial to note that solely the muscle activation gradually increased between each prescribed perceived effort intensity. In experiment 1, our planned follow-up tests on the main effect of effort intensity failed to reveal a significant increase in respiratory frequency between each intensity. These tests also revealed that heart rate frequency solely increased between the intensities moderate to strong, and not between the light to moderate and strong to very strong intensities. As upper-limb motor tasks involve a lower muscle mass than locomotor exercise or resistance exercise and increasing the muscle mass involved in a task is known to increase cardiorespiratory responses to the exercise (Sidhu et al., 2013; MacInnis et al., 2017), the lack of observed increase between intensities in heart rate frequency and respiratory frequency in our study may be due to the low muscle mass involved in the tasks performed. In experiment 2, we used a chest belt to better control movement artifact and increase the quality of our heart rate frequency measurement. Using the chest belt, compared to the finger pulse transducer, allowed us to avoid data loss and capture an increased heart rate frequency between the moderate to strong and strong to very strong intensities, but not between the light to moderate intensities. Consequently, by integrating the two experiments, our results suggest that when prescribing the exercise during upper-limb motor tasks with the intensity of perceived effort, researchers and clinicians should prioritize the use of EMG over heart rate and respiratory frequencies to monitor physiological changes in the physical resources engaged in the task.

### 4.2. Perception of effort changes with the manipulation of physical demand

Perception of effort is not only used to prescribe the exercise but also to monitor the exercise (Borg, 1998; Eston and Parfitt, 2018). Indeed, the intensity of perception of effort during a motor task has been extensively shown to be responsive to changes in task difficulty imposed by various experimental manipulations. As an example, the perception of effort is altered by the intensity of muscle contraction (e.g., de Morree and Marcora, 2010, 2012), the presence of muscle or mental fatigue (e.g., Pageaux and Lepers, 2016, 2018; Jacquet et al., 2021), or changes in environmental conditions (e.g., Girard and Racinais, 2014; Borg et al., 2018; Jeffries et al., 2019). In our study, to test the possibility to monitor the exercise intensity during upper-limb motor tasks, we altered task difficulty by manipulating the physical demand of the tasks performed *via* imposing various movement tempos or adding weights on the forearm. We expected the perception of effort to raise with task difficulty, regardless of the type of physical demand manipulation used.

In experiment 1, during the tempo session, we manipulated the physical demand of the task by imposing three different movement speeds to complete the box and block test and pointing task. The increased number of blocks moved during the box and block test and targets reached during the pointing task confirmed that we were successful in our experimental manipulation. We observed an increased perception of effort between each task difficulty, suggesting the possibility to track changes in task difficulty imposed by changes in movement speed during upper-limb motor tasks. This increased perception of effort was associated with consistently increased muscle activation and heart rate frequency during both tasks. During the weight session, we manipulated the physical demand of the task by adding weights on the forearm and imposing a single movement tempo to constrain performance across task difficulties. The lack of changes in performance in both tasks across difficulties confirms that we were successful in our experimental manipulation. In line with the motivational intensity theory (Brehm and Self, 1989; Richter et al., 2016), when task difficulty increases, performance could be maintained by increasing the effort invested in the task. This proposed mechanism to maintain performance is verified in our experiment *via* the increased perception of effort intensity between each task difficulty, suggesting the possibility to track changes in task difficulty imposed by manipulating the weight of the exercising forearm moved during upper-limb motor tasks. The increased muscle activation and heart rate frequency over task difficulties further support the mechanism proposed by the motivational intensity theory. However, it is noticeable that muscle activation consistently increased between difficulties solely in the biceps brachial muscle and not the triceps brachial muscle. This result suggests that researchers and clinicians interested in monitoring EMG as a physiological marker of perception of effort may prioritize the monitoring of the biceps brachial EMG signal.

In experiment 2, we performed the standardized version of the box and block test by adding a weight on the forearm to increase task difficulty. Neither performance nor movement speed was controlled, the participants had to move as many blocks as possible in 60 s. In this specific experimental paradigm, the motivational intensity theory would predict two possible outcomes (Brehm and Self, 1989; Richter et al., 2016): (i) performance will drop if the increase in task difficulty is beyond the participant’s capacity, or (ii) performance will be maintained if the increase in task difficulty is within the participant’s capacity, and this maintained performance will be possible at a cost of a higher effort invested in the task. As our participants were young and healthy, and the weight added to the forearm was chosen following pilot experiments aiming to limit the development of fatigue, we expected that our participants would be able to maintain performance by increasing the effort invested in the task. In line with our hypothesis performance did not differ between the easy and hard difficulty, and the maintained performance was associated with an increased rating of perceived effort reported by the participants. This increase in perception of effort was associated with increased muscle activation, as observed in experiment 1 to compensate for the heavier forearm to move during the box and block test.

Not all the physiological variables monitored were responsive to changes in task difficulty in both experiments. In experiment 1, the respiratory frequency did not increase between the difficulties easy and medium in both tasks when the physical demand was manipulated with the tempo, and no main effect of task difficulty was observed on respiratory frequency when the physical demand was manipulated with the addition of weight on the forearm. Regarding heart rate frequency, changes in this variable between each difficulty were consistently observed only when the task difficulty was manipulated with the tempo. Furthermore, the increased perception of effort observed in experiment 2 to maintain performance during the box and block test performed with the standardized instructions did not occur in the presence of increased heart rate frequency. These results extend the previous observation of the lack of changes in heart rate frequency and respiratory frequency when the intensity of perceived effort is used to prescribe the exercise and confirm that neither heart rate nor respiratory frequency can be used as an efficient physiological correlate of perception of effort in the context of upper-limb motor tasks. The only parameter responsive to our experimental manipulations was muscle activation, especially biceps brachial muscle activation. Our results suggest that muscle activation of the biceps brachial could be an appropriate physiological marker of the perception of effort during upper-limbs motor tasks. As muscle groups other than the biceps and triceps brachial are involved in the tasks performed, future studies should challenge and extend this observation by measuring activation of other muscle groups during similar tasks (e.g., deltoid muscles). Most likely, the muscles that best quantify effort and correlate with its perception will change with the investigated tasks.

Additionally, it is important to note that we systematically monitored the perceived workload of each task at each difficulty by using the NASA-TLX scale, a validated tool used to monitor perceived workload in various contexts (Hart and Staveland, 1988; Hart, 2006). While this scale captured most manipulations of the physical demand performed in both experiments, a lack of changes in the physical demand score, temporal demand score, or effort score was observed in some experimental conditions. Therefore, our results suggest that the monitoring of the perception of effort with category ratio scales as we did in this study could be a complementary approach for researchers in human factors interested in capturing fine changes in perceived workload when task difficulty is manipulated.

### 4.3. Integration with the neurophysiology of perception of effort

While our experiment did not aim to investigate the neurophysiology of perception of effort, the changes (or lack of changes) in the physiological variables monitored during both experiments allow us to reconcile our results with existing theories on the neurophysiology of perception of effort in the context of motor tasks (de Morree and Marcora, 2015; Pageaux, 2016). In brief, while there is an ongoing debate on the sensory signal(s) generating the perception of effort (Marcora, 2009; Amann and Light, 2014; Smirmaul, 2014; Pageaux, 2016; Broxterman et al., 2018; Steele and Fisher, 2018), accumulating evidence suggests that when effort perception is investigated as a sensation dissociated from other exercise-related sensations (e.g., pain or discomfort), perception of effort is generated by the neuronal process of the corollary discharge associated with the central motor command and not by afferent feedback from the working muscles and organs (Marcora, 2009; de Morree et al., 2012, de Morree and Marcora, 2015; Pageaux and Gaveau, 2016). Our results are consistent with this corollary discharge model of perception of effort. Indeed, muscle activation measured with EMG is traditionally used as a marker of the central motor command (Thoroughman and Shadmehr, 1999; Carrier et al., 2011; Gaveau et al., 2021; Kozlowski et al., 2021), and among the three physiological variables measured, only muscle activation was able to track the changes in perception of effort across manipulations of task difficulties and prescription of exercise *via* the intensity of this perception. Furthermore, in line with the corollary discharge model of perception of effort and the traditional use of this perception as a marker of the central motor command (McCloskey et al., 1974; Mitchell et al., 1989; Kjær et al., 1999; Seed et al., 2019; Jacquet et al., 2021; Kozlowski et al., 2021), our results should motivate the monitoring of this perception in various population with impaired motor control such as older adults (Carment et al., 2018), patients with stroke (Neva et al., 2019), patients with Parkinson’s disease (Sacheli et al., 2019), or other populations with neurological disorders. Future studies should replicate our results with such populations and explore how this perception in the context of specific upper-limb motor tasks is impaired in comparison to healthy individuals. Such studies could provide interesting insights into this perception by further validating its use as a marker of the central motor command in various populations, and potentially open new possibilities in the rehabilitation and testing of capacities.

### 4.4. Limits, perspectives, and conclusion

While our results provide strong support in favor of the use of the perception of effort to prescribe and monitor exercise in the context of upper-limb motor tasks, we have to acknowledge some limitations to be considered for future studies. While our sample size is appropriate for detecting changes associated with moderate to large effect sizes, future studies should increase the sample size and test finer manipulations of the physical demand. Such an increase in sample size and additional manipulations of the physical demand are important next steps to identify the responsiveness of the CR100 scale to measure the perception of effort in the context of upper-limb motor tasks. However, it is important to note that from a clinical perspective, our results replicating moderate to large effects across different experiments are of great importance and should not be neglected. Increasing the sample size could also provide perspectives for investigating sex, gender, and ethnicity differences in the use of the perception of effort to monitor and prescribe exercise. Despite our attempt to control for the induction of fatigue, subjective feelings of fatigue slightly increased in the weight session of experiment 1 (+ 0.9 ± 1.5 on a visual analog scale). However, as the completion of the box and block test and the pointing task, as well as the difficulties, were randomized, we are confident that this slight increase in fatigue did not impact the validity of our results. Nonetheless, future studies using physical demand manipulations and controlling for the presence of fatigue should consider increasing the recovery period between each task completion. In this study, we focused on the box and block test as well as a pointing task, and our results should be extended to other upper-limb tasks routinely used in research and clinical settings with a stronger focus on manual dexterity such as the Purdue pegboard test (Backman et al., 1992; Shahar et al., 1998) or the Minnesota manual dexterity test (Lourenção et al., 2005; Cederlund, 2009). To conclude, this study provides strong evidence in favor of the use of the perception of effort to prescribe and monitor the exercise in the context of upper-limb motor tasks. By integrating the results of the two experiments, measurement of muscle activation, and especially muscle activation of the biceps brachial, seems to be the best physiological correlate of perception of effort during upper-limb motor tasks when the physical demand of the task is manipulated. However, as the muscles that best quantify effort and correlate with its perception will likely change with the investigated tasks, and physiological responses other than muscle activation are likely task-specific, future studies should further explore the identification of psychophysiological correlates of perception of effort in different upper-limb motor tasks. Additionally, the results demonstrating an increased mental demand when physical demand was manipulated with the tempo and weight add to the literature proposing shared mechanisms between physical and mental effort (e.g., Preston and Wegner, 2009). These results reinforce the need for future research challenging the idea that effort perception may encompass both physical and mental aspects of engagement in a task. As effort is perceived not only in the physical domain but also in the mental domain (Preston and Wegner, 2009; Pageaux, 2016; Inzlicht et al., 2018), future studies should test the possibility to extend our results in the context of the manipulation of the mental demand.

## Data availability statement

The original contributions presented in this study are included in the article/Supplementary material, further inquiries can be directed to the corresponding author.

## Ethics statement

The studies involving human participants were reviewed and approved by Local Ethics Committee. The patients/participants provided their written informed consent to participate in this study.

## Author contributions

AC, BP, JG, MG, and PR designed the study. AC, CF-B, and MG conducted the experiments. AC, BP, JG, and MG contributed to the data analysis. MG and BP created the figures. MG created the first draft of the manuscript. All authors edited and/or approved the final version.
