# Supplementary material for: Perception of effort and the allocation of physical resources: A generalization to upper-limb motor tasks
Source: Front Psychol. 2023 Jan 24;13:974172. doi: 10.3389/fpsyg.2022.974172 (PMC9904170; doi:10.3389/fpsyg.2022.974172)

Preliminary experiment to decide which muscle group to use for EMG monitoring.

Twenty participants performed the Box and Block Test in the absence (0 kg) and presence (0.5 kg) of an additional weight over the forearm.

As shown in the results below, the biceps brachial presented the lowest p-values and highest effect size in response to the experimental manipulation. We therefore decided to measure this muscle in the main experiments, as well as its antagonist that best responded to the experimental manipulation (i.e, triceps lateral)

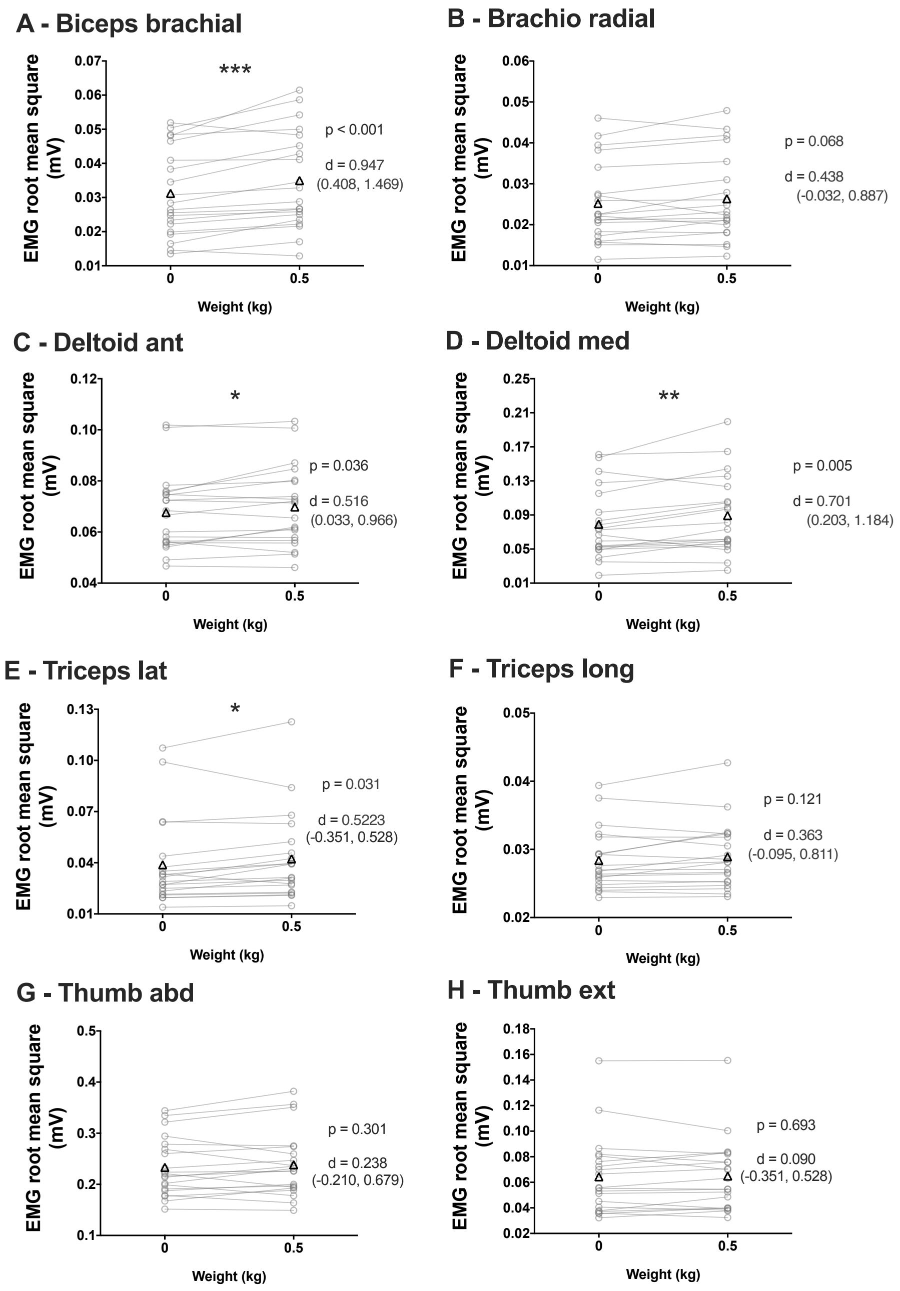

Supplement: Supplementary file 1 [file Data_Sheet_1.pdf]
